# Unmasking Pulmonary Parenchymal Changes in Psoriasis Patients: A Radiological Perspective

**DOI:** 10.3390/medicina61020196

**Published:** 2025-01-23

**Authors:** Müfide Arzu Özkarafakılı, Mustafa İlteriş Bardakçı, Onur Sivaz, İlknur Kıvanç Altunay

**Affiliations:** 1 Department of Chest Diseases, Şişli Hamidiye Etfal Training and Research Hospital, 34381 İstanbul, Turkey; milterisbar@hotmail.com; 2 Department of Dermatology and Venereology, Dörtyol Hatay State Hospital, 31600 Antakya, Turkey; onursivaz@gmail.com; 3 Department of Dermatology and Venereology, Şişli Hamidiye Etfal Training and Research Hospital, 34381 İstanbul, Turkey; ialtunay@gmail.com

**Keywords:** psoriasis, pulmonary involvement, high resolution CT, interstitial changes

## Abstract

*Background and Objectives*: The relationship between psoriasis and pulmonary comorbidities remains to be explained. Our main objective was to investigate pulmonary parenchymal alterations in psoriasis outpatients with chest CT scans who did not exhibit pulmonary symptoms, regardless of their course of treatment or disease severity. *Materials and Methods*: We examined pulmonary function tests, laboratory data, and SF-36 questionnaires from 270 consecutive psoriasis patients who underwent high-resolution computed tomography scans. Psoriasis duration, treatment details, and smoking status were analyzed to identify the associations affecting lung involvement. *Results*: The median age was 48 years, and the median duration of psoriasis was 15 years. A total of 72.6% were on biologics with a median PASI score of 1.5. In total, 43.2% were current smokers. Radiologists reported parenchymal lesions in 118 (43%) of the 270 patients’ HRCT images. Reticular changes (41%) were the most common radiological finding, followed by nodules (38%), and emphysematous changes (21%). Only age, mental health, and smoking status were found to influence the possibility of the occurrence of HRCT findings in multivariate analysis (*p* < 0.001). PASI scores and treatment options did not impact pulmonary parenchymal alterations (*p* > 0.05). *Conclusions*: The striking part was that when compared to never smokers, the imaging findings were 1.9 times more common in current smokers (*p* < 0.05). Using international consensus criteria, two (0.01%) patients were radiologically diagnosed as UIP, and two (0.01%) were identified as NSIP. Psoriasis patients may exert pulmonary disease without clinical manifestation. Pulmonary function tests and radiological evaluation with CT are highly recommended in detecting pulmonary parenchymal changes when indications such as age and current smoking history are present.

## 1. Introduction

Psoriasis is a chronic, inflammatory skin disease affecting more than 125 million patients globally [1]. It is immunologically mediated and highly associated with some well-documented comorbidities like hyperlipidemia, diabetes, obesity, and psychiatric disorders, which suggests that the disease extends outside the limits of the skin [2]. Accumulating data also indicate an increased risk for potential lung involvement in psoriasis. It has been associated with Chronic Obstructive Pulmonary Disease (COPD), asthma, Obstructive Sleep Apnea Syndrome, pulmonary hypertension, sarcoidosis, and interstitial lung disease (ILD) [3,4,5,6,7,8]. In a mouse model study, the increasing count of both neutrophils and eosinophils in the airways implies that psoriasis may increase the airway inflammatory status [9]. Systemic inflammation in psoriasis, higher levels of C-reactive protein, and pro-inflammatory cytokines like interleukin-17, interleukin-23, and interleukin-36 are thought to play a role in both skin and lung inflammation and the airway remodeling pathway [10]. Despite growing evidence that psoriasis and lung involvement are related, little is known about the specific chest computerized tomography (CT) findings of psoriasis because it is not regularly investigated unless respiratory symptoms become apparent [7,8]. In several reports, ILD in psoriasis patients has been defined mostly as drug-induced pneumonitis triggered by immunosuppressants and biologics; however, the coexistence of interstitial pneumonia and psoriasis has also been documented in recent studies [8]. In this context, we aimed to examine the psoriasis patients’ pulmonary function tests, radiological results, blood parameters, and quality of life surveys. We intended to determine whether this relationship, if any, connects to the severity of the disease, available treatments, and pulmonary parenchymal alterations observed on chest CT scans.

## 2. Material and Methods

This single-center, cross-sectional study was conducted in a tertiary care hospital after the institutional ethics committee approved it. The study included psoriasis patients aged 18 and older admitted to the Dermatology Outpatient Clinic consecutively between March 2022 and December 2024. The patients in the study have cutaneous psoriasis, either receiving systemic therapy or phototherapy. Psoriasis was diagnosed as a systemic inflammatory disease characterized by cutaneous erythema and macroscopically visible sterile pustules [1]. Patients who were pregnant, had autoimmune diseases, cancers, uncontrolled cardiovascular disease, collagen vascular disease, active pulmonary symptoms from respiratory tract infections or a prior diagnosis of lung disease, occupational dust exposure, or had neurological insufficiency that could affect their quality of life were excluded.

A total of 270 patients with psoriasis who were referred to the outpatient department of Chest Disease and had a high-resolution computerized tomography scan (HRCT) of the chest for some purposes were found eligible for the study. HRCT is carried out under full inspiration and yields thin-section CT images with a 1 mm slice thickness. The HRCT scans were all reported by two experienced thoracic radiologists.

The HRCT findings were characterized as nodule (a circumscribed, typically round opacity, less than or equal to 30 mm in average diameter), reticular changes (collection of intersecting linear opacities that produce an appearance resembling a net), ground-glass opacity (area of increased attenuation that does not completely obscure the underlying bronchial and vascular structures), mosaic changes (patchwork of regions with different attenuation), emphysema (centrilobular, areas of low attenuation surrounded by normal lung, without clearly defined walls; panlobular, large or diffuse areas of low attenuation, where components of the normal lung architecture, and notably those of the secondary pulmonary lobule, are no longer recognizable; or paraseptal, foci of low attenuation separated by intact interlobular septa typically located near the pleural surface close to the chest wall, the mediastinum, and interlobar fissures), and bronchiectasis (dilatation of the bronchus relative to the accompanying pulmonary artery, lack of tapering, and presence of visible bronchi within 1 cm of the pleural surface; bronchial walls can be thickened) [11]. In addition, there were fibrotic lines and calcified nodules in CT scans.

The patients were categorized into three groups based on their Psoriasis Area Severity Index (PASI) scores, which indicated the degree and severity of the disease: mild (<5), moderate [5,6,7,8,9,10], and severe (>10). Information about smoking habits, medical history for coexisting conditions, treatment details, demographics, and the length of the illness were all documented.

Initially, we performed a pulmonary function test (PFT) according to the American Thoracic Society criteria [12]. Predicted % values of forced expiratory volume in the 1st second (FEV_1_) and forced vital capacity (FVC) were measured and the FEV_1_/FVC % ratio was calculated.

We queried about the patient’s general quality of life using the Short Form-36 (SF-36) in Turkish. It is a health survey that consists of 36 questions and has been validated for the Turkish population [13]. The physical and mental health scores ranged from 0 to 100, with higher values indicating a higher quality of life.

All the participants’ venous blood samples were collected in order to measure the complete blood count and C-reactive protein.

Finally, the demographic data, clinical characteristics, pulmonary function test results, and radiological findings of the 270 patients were evaluated and intergroup comparison was performed regarding the presence of HRCT findings.

### Statistical Analysis

Patient data collected within the scope of the study were analyzed with the IBM Statistical Package for the Social Sciences (SPSS) for MacOS 29.0 (IBM Corp, Armonk, NY, USA) package program. Frequency and percentage were provided for categorical data, while median, minimum, and maximum descriptive values were provided for continuous data.

Group comparisons were conducted using the “Mann–Whitney U Test” for two groups, the “Kruskal–Wallis H Test” for more than two groups, and the “Chi-square or Fisher’s Exact Test” for categorical variable comparisons.

In the distribution of the demographic, clinical, and radiological findings of the patients according to treatment options and smoking status, the Mann–Whitney U Test with Bonferroni correction was performed to determine which groups were responsible for the significant difference in the variables that showed significant differences as a result of the Kruskal–Wallis H Test. A logistic regression analysis was also used to determine the risk factors affecting the development of findings in the HRCT scan. In the multivariate model, the most appropriate model was determined using the retrospective Wald method. The results were considered statistically significant if the *p*-value was less than 0.05.

## 3. Results

Table 1 summarizes the patients’ demographic features, clinical presentation, SF-36 ratings, spirometry, and laboratory findings. The study comprised 270 consecutive patients, including 119 females (44.1%) and 151 males (55.9%). Their ages were between 18 and 76 years, with a median of 48 years. The median duration of the disease at the time of pulmonary evaluation was 15 years.

The median body mass index (BMI) was 27.1 (range 17.28–49.7). The current treatments were as follows: 196 (72.6%) on biologics, 54 (20%) on methotrexate, 15 (0.05%) on acitretin, 3 (0.01%) on cyclosporine, and 2 (0.007%) on phototherapy.

A total of 41 (15.2%) patients had cardiovascular disease, 31 (11.5%) had depression, 32 (11.9%) had diabetes, 27 (10%) had arthritis, and 8 (3%) had nonalcoholic fatty liver disease. In total, 102 (37.8%) of them were current smokers.

The median predicted % values for the spirometry measures were 89 (range 24–140) for FEV_1_, 89 (range 39–133) for FVC, and 102 (range 64–127) for the FEV_1_/FVC ratio.

In total, 28 (10%) individuals were diagnosed with COPD based on a combination of medical history, physical examination, and pulmonary function tests; a post-bronchodilator FEV_1_/FVC% ratio < 70 and a FEV_1_% < 80 were accepted as indications of airflow restriction [12].

According to the GINA criteria, 27 (10%) patients with pulmonary function tests showing a 12% and 200 mL forced expiratory volume in 1 s (FEV_1_) reversibility following inhalation of 400 mg salbutamol were diagnosed with asthma [14]. CT abnormalities have been detected in 118 (43%) of these 270 individuals.

The study participants were grouped based on the presence of findings in HRCT. The median age for the patients who had findings on their HRCT scan was 57 years, the duration of psoriasis was 16 years, and the PASI score was two.

In comparison to the patient group without HRCT findings, the patient group with HRCT findings had statistically higher levels of age, BMI, disease duration, PASI scores, participant smoking rates, diabetes mellitus, depression, and cardiovascular disease (*p* < 0.005). The FEV1% value had also been statistically lower in the patient group with HRCT findings (*p* < 0.005). Current smokers accounted for 43.2%, while former smokers accounted for 19.5% of the patients with radiological abnormalities on an HRCT scan. Additionally, 60.2% of these patients had comorbidities and 33.1% had cardiovascular disease, and 16.9% had depression and diabetes.

A statistically significant difference was found between the two groups in terms of health quality indicators such as physical and emotional role difficulty scores, mental health scores, and pain scores (*p* < 0.05). Although the median values of physical and emotional role difficulty scores and mental health scores were comparable in the groups, it was observed that the average scores were higher in individuals without HRCT findings than in those with HRCT findings.

The distribution of the demographic and clinical findings of the patients according to the treatments they received within the scope of the study is shown in Table 2.

When we examined the patients based on the treatment options, we found that the group treated with medications other than methotrexate or biological agents had higher age and PASI scores, lower FEV_1_% values, and lower rates of comorbidity (*p* < 0.005). The group of patients on biological agents was younger, and their PASI scores were lower, but the duration of the disease was the longest, and the rates of having a comorbidity were highest in that group compared to the patients on methotrexate and other medications (*p* < 0.005). Cardiovascular diseases, depression, and arthritis were more common among the patients on biological agents (*p* < 0.005). According to the available treatment options, there was no difference in the groups’ gender distribution, BMI, laboratory results, smoking status, or the existence of HRCT findings (*p* > 0.005).

The radiological features of lung parenchyma were categorized as previously mentioned. The radiological abnormalities that were most frequently observed were reticular changes (41%). The other findings detected on HRCT were nodules in 38%, emphysematous changes in 21%, fibrotic lines in 18%, ground-glass opacities in 16%, mosaic changes in 13%, bronchiectasis in 10%, and calcified nodules in 0.05% of the patients.

Table 3 depicts the distribution of the risk factors that influence the occurrence of HRCT findings in our study’s subjects. According to univariate analysis, the following factors influenced the occurrence of HRCT findings: age, psoriasis duration, FEV_1_% value, physical and emotional role difficulties, mental health, pain, smoking status, presence of comorbidities, diabetes mellitus, depression, and cardiovascular diseases (*p* < 0.05).

It was observed that each unit increase in age (*p* < 0.001) and duration of psoriasis (*p* = 0.004) caused an increasing effect in the occurrence of HRCT findings, while each unit increase in FEV_1_% value (*p* = 0.008), physical role difficulties (*p* < 0.001), emotional role difficulties (*p* < 0.001), mental health (*p* = 0.004) and pain (*p* = 0.022) parameters caused a decreasing effect. The HRCT findings were 1.9 times more common in the current smokers than in the never smokers, and 2.8 times more common in the former smokers. Also, it was found that the risk is higher in the patients with comorbidities (*p* < 0.001), diabetes mellitus (*p* = 0.025), depression (*p* = 0.016), and cardiovascular disease (*p* < 0.001) than in those without.

When the parameters with significant differences in univariate analysis were re-evaluated using the retrospective Wald method in multivariate analysis, it was found that age (*p* < 0.001), mental health score (*p* = 0.048), and current smoking (*p* = 0.015) were the factors affecting the occurrence of HRCT findings.

Table 4 displays the distribution of patients’ demographic, clinical, and HRCT data based on their smoking status. Former smokers were found to be older than never smokers, and having a comorbidity was more common in currently smoking patients than in never smokers (*p* < 0.05). Although no statistical difference was found, cardiovascular disease, depression, and diabetes were more common among the current smokers (*p* = 0.05). There were no differences in gender, psoriasis duration, PASI scores, pulmonary function tests, or laboratory findings between the patient groups depending on their smoking status (*p* > 0.05). The presence of HRCT findings was more frequent in the current smokers and when compared to the former and never smokers, emphysematous changes were statistically significantly higher in the current smokers (*p* < 0.05). In addition, bronchiectasis was more common among the former smokers than the current and never smokers (*p* < 0.05). Reticular changes (22.5%) and nodules (18.6%) were found to be the most common findings in the currently smoking patients.

A total of 68 (57.6%) of the patients had only one, whereas 5 (0.04%) had more than three radiological findings on the HRCT scan. The reported nodules were <6 mm in diameter, and the fibrotic lines and calcified nodules were identified as pleuroparenchymal sequelae of tuberculosis. Based on the HRCT patterns, two (0.01%) patients were radiologically diagnosed as UIP (usual interstitial pneumonia), and two (0.01%) patients were as NSIP (Non-specific Interstitial Pneumonia) using international consensus criteria [15].

## 4. Discussion

Lung disorders and their associations with psoriasis have received less attention despite prior studies demonstrating abnormalities in pulmonary function and radiographic signs of parenchymal alterations in these patients [16,17]. The most important implication of our study was the detection of interstitial and parenchymal changes in various combinations in HRCT scans performed during the systemic evaluation of psoriasis patients. Age and lifetime smoking consistently influenced the HRCT findings’ occurrence, whereas therapeutic agents, laboratory parameters, duration of psoriasis, and disease severity did not appear to have a statistically significant impact.

A growing body of evidence indicates that psoriasis is a chronic condition, and its systemic component might lead to several disorders such as psoriatic arthritis, diabetes, depressive symptoms, cardiovascular events, chronic fatty liver disease, and metabolic syndrome [2]. Besides these well-known comorbidities, recent research has reported subclinical lung involvement suggesting that psoriasis may be accompanied by a mild and slowly progressive form of interstitial lung disease [17,18]. In a very recently published article, researchers defined ILD among patients with plaque psoriasis who even did not require systemic treatment [19]. That may constitute the new approach for an association of ILD and psoriasis with the autoimmune nature of pathogenesis [8]. Several complicating variables may influence these individuals’ lung parenchyma, including frequent infections and potential pulmonary adverse effects from various therapeutic agents. These drugs are utilized throughout the illness course and are associated with ILD in psoriasis cases [19]. As a result, the data on pulmonary comorbidities in psoriasis revealed inconsistent conclusions.

A univariable analysis revealed that BMI, disease duration, FEV_1_% values, and the existence of diabetes or cardiovascular problems all influenced the presence of HRCT findings in our study. However, after adjusting for confounding variables, only age and current smoking were found to be effective in detecting radiographic lung lesions in multivariable analyses.

Furthermore, these CT findings were not associated with either methotrexate or biological agent use or psoriatic arthritis and were not accompanied by manifest respiratory symptoms. The main manifestation of interstitial findings in our study was reticular changes (41%), followed by fibrotic lines (18%), ground glass opacities (16%), mosaic attenuation (11%), or a combination of these patterns.

The majority of these lesions were focal, mild, and patchy interstitial changes that involved the subpleural zone. These incidentally found imaging features at CT might represent the interstitial lung abnormalities (ILAs) or ILA-like appearances reported by the Fleischner Society of Radiology [20]. The clinical significance of these findings is that they may progress in about half of the patients within 5 years and eventually develop into pulmonary fibrosis [21]. The prevalence of these abnormalities is pointed out at 4–7% in smokers. Aging and tobacco smoke are defined as major risk factors for the development of these interstitial changes. In a screening context, ILA was detected in 194 (8%) of a group of smokers’ high-resolution CT scans [22].

Furthermore, the previously mentioned investigations demonstrate a correlation between subpleural reticular changes and lower lobe predominancy with the progression and early indicators of pulmonary fibrosis, underscoring the significance of our results in this screening context. Several reports revealed that aging-related alterations have been linked to the development of idiopathic pulmonary fibrosis [21,22].

Nevertheless, among the participants of our study, there were two (0.01%) patients radiologically diagnosed with nonspecific usual interstitial pneumonia (NSIP) and two (0.01%) patients with usual interstitial pneumonia (UIP) patterns. In both cases, the lesions were predominantly located peripherally and involved the lower lobes of the lung. In their psoriasis group, Ishikawa and Kawamoto et al. reported an incidence of ILD of 4.7% and 2%, respectively, which was higher than the results of our study [8,19]. It is worth mentioning that different from our study, 63.6% of the psoriasis patients with ILD in Ishikawa et al.’ s and 32% in Butt et al.’s study consisted of treatment-naïve patients [8,23]. Kawamoto’s ILD patients primarily had ground glass and linear opacities on CT scans and did not exhibit any respiratory symptoms like our patients did. However, their psoriasis cohort was on biologics with severe disease (PASI > 10), while ours had mild disease (PASI < 5), and almost 70% of our patients were prescribed biologics [19]. In a recent study, researchers reported higher rates of UIP patterns among patients with psoriasis who were smokers in a lifetime than those who never smoked [24].

A total of 43.2% of our patients with HRCT findings were actively smoking individuals. According to the latest data, that was significantly higher than the prevalence of Turkish smokers, which was estimated at 31.2% among those over the age of 15 [25]. Although we found smoking effective in the appearance of CT findings, we noted no significant differences between never smokers and smokers regarding the characteristics of the interstitial changes (*p* > 0.05). Emphysematous changes (21%) were prevalent among the current smokers, indicating that these individuals were candidates for the development of COPD, and 10% of our research participants exhibited airway obstruction on pulmonary function testing.

Nodules < 6 mm in diameter (38%) were the second most common radiological features after reticular changes in our whole study group. Samrah et al. found nodules in 66.1% of their study group’s HRCT scans who had psoriasis [17]. These incidences were much higher than those reported in research conducted in the United States, which found 6.6 lung nodules per 1000 individuals [26].

Bronchiectasis was detected in 10% of our patients, which might be a sequela of a recent or previous infection. Fibrotic lines, ground glass opacities, and mosaic changes were found in 18%, 16%, and 11% of our patients’ CT scans, respectively; nevertheless, most of the lesions were focal, mild, and patchy interstitial changes that involved the subpleural zone.

There was no correlation between the existence of HRCT findings and the PASI score, CRP an indicator of systemic inflammation, treatment status, or the SF-36 parameters reflecting the quality of life (*p* > 0.05). Parallel to our results, in a USA-based study, the SF-36 scores in individuals with mild to severe psoriasis patients were found to be near normal compared to the US population [27].

The CRP levels change over time depending on treatment status, and in mild cases as in our group, they tend to be in the normal range in line with the literature [27]. The ILD patients with psoriasis in Matsumoto’s study were also mildly symptomatic, raising the possibility that these interstitial changes might be different from the conventional pulmonary fibrosis course [28]. This stemmed from the hypothesis that prolonged low-grade systemic inflammation may generate vascular disorders in psoriasis [29]. The high amounts of circulating inflammatory biomarkers, such as CRP and IL-22, which were streamed from skin lesions into the bloodstream, may serve to explain the association between the severity of psoriasis and respiratory disorders [30].

Although epidemiological studies show a low incidence of interstitial lung disease (ILD) associated with psoriasis, this topic has begun to draw attention in recent studies and case reports [22,31]. Researchers discovered in a different study that individuals with psoriasis were more likely to acquire sarcoidosis than controls [7]. In addition, methotrexate and some biological agents prescribed for psoriasis may potentially trigger interstitial lung disease, and ILD was occasionally reported in these individuals, but the causality was unclear [32]. According to reports, type 17 helper T cells are one of the key components of pathophysiology in psoriasis, which are also known to be involved in the mechanisms of alveolitis and enhancing cytokine production in pulmonary fibroblasts [30]. IL 17 A, 17 F, and 22, which were produced by activated type 17 helper cells, have been found to induce keratinocyte proliferation in the skin, and might play a role in concurrent pulmonary fibrosis in the lungs [33]. Another study revealed that skin cell proliferation in psoriasis, which was secondary to activated Tumor Growth Factor-alpha (TGF-α), might also be connected to TGF-β related pulmonary fibrosis [34,35]. The IL-17/IL-23 axis seems to be involved in the pathogenesis of both psoriasis and lung fibrosis; also, biological agents inhibiting that pathway have been shown to improve both skin and lung lesions in some studies [30,35]. Psoriasis-induced immune dysfunction may result in an aberrant immune response in the lung parenchyma due to its potential autoimmune-related etiology [8].

It is worth noting that the duration of psoriasis disease in our study (median 16 years) was longer than in the abovementioned studies, which might explain the parenchymal changes in our patients’ lungs. Furthermore, the difference in pulmonary function tests based on the presence of HRCT findings was significant but mild in our patients (FEV_1_ 89% versus 88%, FVC 90% versus 89%, and FEV_1_/FVC 103% versus 101%, respectively), indicating a modest form of interstitial lung pattern compared to conventional idiopathic pulmonary fibrosis. Although inflammatory-based comorbidities such as diabetes, depression, and cardiovascular diseases were risk factors for the development of ILD, no association was found between the comorbidities and the presence of parenchymal changes in our patients (*p* > 0.05) [36].

We speculated that immunological dysfunction in psoriasis patients might also contribute to low-grade inflammation and lead to fibrosis in the lungs in the late stages of the disease. The relationship between psoriasis and lung diseases is not fully understood, and it has not yet been determined whether psoriasis itself poses a risk for pulmonary diseases. Because not all of the earlier investigations employed HRCT scans, the data were underpowered, and the conclusions were debatable.

## 5. Conclusions

The study had several limitations: it was conducted in a single center and limited only to patients with HRCT scans; therefore, the results may not be generalizable. The diffusing capacity for carbon monoxide tests, which was an important aspect of evaluating pulmonary functioning in interstitial lung disease, was unavailable.

However, it is crucial to recognize the imaging findings, and our results suggest that the possible existence of an association between psoriasis and lung involvement seems plausible. Our study advocates approaching psoriasis as an autoimmune disease and considering the presence of interstitial changes associated with psoriasis.

Interstitial lung disease in psoriasis is a new venue for researchers. This study mainly highlighted the awareness of pulmonary parenchymal changes in psoriasis patients even with no respiratory manifestations since we discovered a higher-than-expected incidence of interstitial changes in their HRCT scan. Pulmonary function tests and HRCT scans serve as easy and useful screening tools in smoker psoriasis patients to determine early pulmonary involvement. Further research in psoriasis patients may be beneficial in determining the common immune dysfunction route and inflammation-based underlying processes of the co-occurring diseases.

## Figures and Tables

**Table 1:** Distribution of demographic and clinical findings of patients according to HRCT findings.

Variables	HRCT Findings			
	Total (*n* = 270)	Absent (*n* = 152)	Present (*n* = 118)	*p*-Value
	*n* (%) or Median (Min–Max)	*n* (%) or Median (Min–Max)	*n* (%) Veya Median (Min–Max)	
Age (years)	48 (18–76)	38 (18–68)	57 (20–76)	<0.001
Gender				0.084
Female	119 (44.1)	60 (39.5)	59 (50)	
Male	151 (55.9)	92 (60.5)	59 (50)	
BMI (kg/m^2^)	27.1 (4.8–49.7)	26.6 (18.8–46.8)	28.7 (4.8–49.7)	0.024
Duration of the disease (years)	15 (0.5–62)	15 (2–42)	16 (0.5–62)	0.048
PASI scores	1.5 (0–31.2)	1 (0–22)	2 (0–31.2)	0.016
Pulmonary function tests				
FEV_1_%	89 (24–140)	89 (67–123)	88 (24–140)	0.015
FVC%	89 (39–933)	90 (70–933)	89 (39–128)	0.109
FEV_1_/FVC %	102 (64–127)	103 (81–127)	101 (64–126)	0.221
SF-36				
Physical functioning	85 (50–100)	85 (50–100)	85 (50–100)	0.359
Physical role difficulties	100 (0–100)	100 (0–100)	100 (0–100)	<0.001
Emotional role difficulties	100 (0–100)	100 (0–100)	100 (0–100)	<0.001
Energy–vitality	70 (25–100)	70 (25–75)	70 (35–100)	0.229
Mental health	72 (48–88)	72 (48–80)	72 (48–88)	0.035
Social functioning	87.5 (50–100)	87.5 (50–100)	87.5 (62.5–100)	0.050
Pain	90 (30–100)	100 (45–100)	77.5 (30–100)	0.013
General health	55 (30–75)	55 (30–70)	55 (30–75)	0.151
Smoking status				0.006
Never smoker	129 (47.8)	85 (55.9)	44 (37.3)	
Current smoker	102 (37.8)	51 (33.6)	51 (43.2)	
Former smoker	39 (14.4)	16 (10.5)	23 (19.5)	
Laboratory				
WBC	7.7 (3.7–13.8)	7.8 (4.4–13.8)	7.7 (3.7–12.8)	0.624
Hematocrit	41 (31.8–49.1)	40.7 (32.6–49.1)	41 (31.8–46.7)	0.584
CRP	1.7 (0–45.2)	1.5 (0–18.6)	1.9 (0–45.2)	0.569
Comorbidities	110 (40.7)	39 (25.7)	71 (60.2)	<0.001
Diabetes mellitus	32 (11.9)	12 (7.9)	20 (16.9)	0.036
Depression	31 (11.5)	11 (7.2)	20 (16.9)	0.022
Chronic liver disease	8 (3)	5 (3.3)	3 (2.5)	1.000
Cardiovascular disease	41 (15.2)	2 (1.3)	39 (33.1)	<0.001
Arthritis	27 (10)	14 (9.2)	13 (11)	0.775
Treatment				0.262
Methotrexate	54 (20)	33 (21.7)	21 (17.8)	
Biological agents	196 (72.6)	111 (73)	85 (72)	
Others	20 (7.4)	8 (5.3)	12 (10.2)	

**Table 2:** Distribution of demographic and clinical findings of patients according to their treatments.

Variables (*n* = 270)	Treatment				
	Methotrexate (*n* = 54)	Biological Agents (*n* = 196)	Other * (*n* = 20)	*p*-Value	Difference **
	*n* (%) or Median (Min–Max)	*n* (%) or Median (Min–Max)	*n* (%) or Median (Min–Max)		
Age (years)	47.5 (20–71)	46.5 (18–76)	56.5 (22–69)	0.019	1–3, 2–3
Gender				0.206	
Female	29 (53.7)	80 (40.8)	10 (50)		
Male	25 (46.3)	116 (59.2)	10 (50)		
BMI kg/m^2^	26.9 (18.1–46.8)	27 (4.8–49.7)	30 (20.3–44.4)	0.052	
Duration of disease (years)	11 (0.5–59)	16 (2–62)	9.5 (2–42)	0.015	2–3
PASI scores	2.5 (0–22)	1 (0–31.2)	4 (0–16.3)	<0.001	1–2, 2–3
Mild	38 (70.4)	175 (89.3)	13 (65)	0.001	
Moderate	11 (20.4)	12 (6.1)	5 (25)		
Severe	5 (9.3)	9 (4.6)	2 (10)		
Pulmonary function tests					
FEV_1_%	88.5 (54–118)	89 (24–140)	85 (31–102)	0.084	
FVC%	90 (53–111)	89 (39–933)	84 (45–102)	0.049	1–3
FEV_1_/FVC %	103 (79–121)	101 (64–127)	100.5 (71–116)	0.650	
HRCT findings	21 (38.9)	85 (43.4)	12 (60)	0.262	
Nodule	10 (18.5)	31 (15.8)	4 (20)	0.821	
Reticular changes	10 (18.5)	34 (17.3)	5 (25)	0.697	
Ground glass opacities	5 (9.3)	12 (6.1)	2 (10)	0.629	
Mosaic changes	3 (5.6)	9 (4.6)	1 (5)	0.957	
Emphysematous changes	3 (5.6)	19 (9.7)	3 (15)	0.425	
Bronchiectasis	2 (3.7)	10 (5.1)	0 (0)	0.549	
Fibrotic lines	3 (5.6)	15 (7.7)	4 (20)	0.116	
Calcified nodule	0 (0)	5 (2.6)	1 (5)	0.361	
Laboratory findings					
WBC	7.6 (4.4–12.8)	8 (4.4–13.8)	7.8 (3.7–11.6)	0.647	
Hematocrit	40.7 (36.3–47.5)	41 (31.8–49.1)	40.4 (35.9–45.9)	0.225	
CRP	1.9 (0–18.6)	1.6 (0–45.2)	2.2 (0–8.1)	0.477	
Comorbidities	25 (46.3)	69 (35.2)	16 (80)	<0.001	
Diabetes mellitus	6 (11.1)	24 (12.2)	2 (10)	0.940	
Depression	6 (11.1)	19 (9.7)	6 (30)	0.025	
Chronic liver diseases	4 (7.4)	4 (2)	0 (0)	0.086	
Cardiovascular diseases	8 (14.8)	25 (12.8)	8 (40)	0.005	
Arthritis	11 (20.4)	13 (6.6)	3 (15)	0.009	
Smoking status				0.109	
Never smoker	30 (55.6)	90 (45.9)	9 (45)		
Current smoker	22 (40.7)	71 (36.2)	9 (45)		
Former smoker	2 (3.7)	35 (17.9)	2 (10)		

* acitretin, phototherapy, and cyclosporin. ** Bonferroni correction was applied for multiple testing.

**Table 3:** Examination of the risk factors affecting the presence of HRCT findings in the patients with psoriasis.

Variables	Univariate Analysis		Multivariate Analysis *	
	Odds Ratio (95% CI)	*p*-Value	Odds Ratio (95% CI)	*p*-Value
Age (years)	1.24 (1.18–1.31)	<0.001	1.25 (1.18–1.32)	<0.001
BMI (kg/m^2^)	1.05 (1.01–1.10)	0.027		
Duration of psoriasis(years)	1.03 (1.01–1.05)	0.004		
PASI scores	1.04 (0.98–1.10)	0.177		
FEV_1_%	0.98 (0.96–0.99)	0.008		
Physical role difficulties	0.98 (0.97–0.99)	<0.001		
Emotional role difficulties	0.98 (0.97–0.99)	<0.001		
Mental health	0.96 (0.93–0.99)	0.004	0.95 (0.91–1.00)	0.048
Pain	0.98 (0.97–0.99)	0.022		
Smoking status				
Never smoker	Reference		Reference	
Current smoker	1.93 (1.14–3.29)	0.015	2.70 (1.21–6.00)	0.015
Former smoker	2.78 (1.33–5.79)	0.006	1.54 (0.49–4.87)	0.463
Methotrexate	0.78 (0.42–1.44)	0.426		
Biological agents	0.95 (0.56–1.63)	0.856		
Comorbidities	4.38 (2.61–7.35)	<0.001		
Diabetes mellitus	2.38 (1.11–5.10)	0.025		
Depression	2.62 (1.20–5.70)	0.016		
Cardiovascular disease	37.03 (8.71–157.35)	<0.001		

* retrospective Wald method.

**Table 4:** Distribution of demographic, clinical, and radiological findings of patients according to smoking status.

Variables (*n* = 270)	Smoking Status				
	Never Smoker (*n* = 129)	Current Smoker (*n* = 102)	Former Smoker (*n* = 39)	*p*-Value	Difference **
	*n* (%) or Median (Min–Max)	*n* (%) or Medyan (Min–Max)	*n* (%) or Medyan (Min–Max)		
Age (years)	45 (18–76)	48 (20–71)	54 (24–71)	0.026	1–3
Gender				0.551	
Female	61 (47.3)	43 (42.2)	15 (38.5)		
Male	68 (52.7)	59 (57.8)	24 (61.5)		
Duration of psoriasis (years)	15 (2–62)	15 (0.5–62)	17 (2–40)	0.855	
PASI scores	1 (0–22)	1.5 (0–17.5)	1.5(0–31.2)	0.437	
Pulmonary Function Tests					
FEV_1_%	89 (31–127)	89 (24–124)	90 (39–140)	0.231	
FVC%	89 (45–128)	89 (39–117)	90 (46–127)	0.575	
FEV_1_/FVC %	102 (71–127)	101 (64–121)	103 (68–116)	0.312	
Comorbidities	40 (31)	53 (52)	17 (43.6)	0.005	
Diabetes mellitus	12 (9.3)	14 (13.7)	6 (15.4)	0.447	
Depression	11 (8.5)	16 (15.7)	4 (10.3)	0.230	
Chronic liver disease	2 (1.6)	6 (5.9)	0 (0)	0.078	
Cardiovascular disease	15 (11.6)	20 (19.6)	6 (15.4)	0.244	
Arthritis	13 (10.1)	12 (11.8)	2 (5.1)	0.501	
Labaratory findings					
WBC	8 (3.7–12.1)	7.7 (4.5–13.8)	7.7 (4.4–9.9)	0.604	
Hematocrit	41 (32.6–48.4)	40.9 (34.7–49)	41.4 (31.8–49.1)	0.791	
C-reactive protein	1.6 (0–45.2)	1.7 (0–18.4)	1.9 (0–14.6)	0.760	
Treatment				0.109	
Methotrexate	30 (23.3)	22 (21.6)	2 (5.1)		
Biological agents	90 (69.8)	71 (69.6)	35 (89.7)		
Other *	9 (7)	9 (8.8)	2 (5.1)		
HRCT findings	44 (34.1)	51 (50)	23 (59)	0.006	
1	26 (59.1)	28 (54.9)	14 (60.9)	0.633	
2–3	16 (36.4)	22 (43.1)	7 (30.4)		
>3	2 (4.5)	1 (2.0)	2 (8.7)		
Nodule	17 (13.2)	19 (18.6)	9 (23.1)	0.277	
Reticular changes	20 (15.5)	23 (22.5)	6 (15.4)	0.343	
Ground glass opacities	7 (5.4)	7 (6.9)	5 (12.8)	0.285	
Mosaic changes	5 (3.9)	5 (4.9)	3 (7.7)	0.621	
Emphysematous changes	5 (3.9)	15 (14.7)	5 (12.8)	0.013	
Bronchiectasis	1 (0.8)	5 (4.9)	6 (15.4)	<0.001	
Fibrotic lines	10 (7.8)	8 (7.8)	4 (10.3)	0.873	
Calcified Nodule	3 (2.3)	2 (2.0)	1 (2.6)	0.971	

* acitretin, phototherapy, and cyclosporine. ** Bonferroni correction was applied for multiple testing.

## Data Availability

The datasets generated during the current study are available from the corresponding author upon reasonable request.
